# To Align Multimodal Lumbar Spine Images via Bending Energy Constrained Normalized Mutual Information

**DOI:** 10.1155/2020/5615371

**Published:** 2020-07-10

**Authors:** Shibin Wu, Pin He, Shaode Yu, Shoujun Zhou, Jun Xia, Yaoqin Xie

**Affiliations:** ^1^Shenzhen Institutes of Advanced Technology, Chinese Academy of Sciences, Shenzhen 518055, China; ^2^Department of Radiology, Shenzhen Second People's Hospital, The First Affiliated Hospital of Shenzhen University, Shenzhen 518035, China; ^3^National Cancer Center/National Clinical Research Center for Cancer/Cancer Hospital & Shenzhen Hospital, Chinese Academy of Medical Sciences and Peking Union Medical College, Shenzhen 518116, China; ^4^Department of Radiation Oncology, University of Texas, Southwestern Medical Center, Dallas, TX 75390, USA

## Abstract

To align multimodal images is important for information fusion, clinical diagnosis, treatment planning, and delivery, while few methods have been dedicated to matching computerized tomography (CT) and magnetic resonance (MR) images of lumbar spine. This study proposes a coarse-to-fine registration framework to address this issue. Firstly, a pair of CT-MR images are rigidly aligned for global positioning. Then, a bending energy term is penalized into the normalized mutual information for the local deformation of soft tissues. In the end, the framework is validated on 40 pairs of CT-MR images from our in-house collection and 15 image pairs from the SpineWeb database. Experimental results show high overlapping ratio (in-house collection, vertebrae 0.97 ± 0.02, blood vessel 0.88 ± 0.07; SpineWeb, vertebrae 0.95 ± 0.03, blood vessel 0.93 ± 0.10) and low target registration error (in-house collection, ≤2.00 ± 0.62 mm; SpineWeb, ≤2.37 ± 0.76 mm) are achieved. The proposed framework concerns both the incompressibility of bone structures and the nonrigid deformation of soft tissues. It enables accurate CT-MR registration of lumbar spine images and facilitates image fusion, spine disease diagnosis, and interventional treatment delivery.

## 1. Introduction

Spine is the backbone of body trunk. It protects the most significant nerve pathway in the spinal cord and the body. On the other hand, spine injury and disorders affect up to 80% world population and may cause deformity and disability, which become a major health and social problem [1–3]. For instance, the lumbar degenerative disease accompanied by pathological changes might result in lumbocrural pain, neural dysfunction, instability of facet joints, and spino-pelvic sagittal imbalance, and thus, the quality of life decreases dramatically. In addition, due to the aging population, the global burden relating to spinal disease remedy is expected to raise significantly in the next decades.

To align intrapatient multimodal images, such as computerized tomography (CT) and magnetic resonance (MR), benefits clinical diagnosis, treatment planning, and delivery for lumbar spinal diseases [4, 5]. However, few methods were dedicated to matching lumbar spine images. Panigrahy et al. developed a method for CT-MR cervical spine images which needed anatomical landmarks to guide image registration [6]. Palkar and Mishra combined different orthogonal wavelet transforms with various transform sizes for CT-MR spine image fusion, while interactive localization of control points was required [7]. Tomazevic et al. implemented an approach for rigid alignment of volumetric CT or MR to X-ray images [8]. To simplify the registration problem in real-world scenarios, images were acquired from a cadaveric lumbar spine phantom and three-dimensional (3D) images contained only one of the five vertebrae. Otake et al. proposed a registration method for 3D and planar images which was used for spine intervention and vertebral labeling in the presence of anatomical deformation [9]. Harmouche et al. designed an articulated model for MR and X-ray spine images [10]. Hille et al. presented an interactive framework, and rough annotation of the center regions in different modalities was used to guide the registration [11].

Accurate alignment of intrapatient CT-MR images is challenging. From the anatomy, human spine consists of inflexible vertebrae surrounded by soft tissues, such as nerves, vessels, and muscles. Moreover, the vertebrae of lumbar spine are connected by facet joints in the back, which allows for forward and backward extension and twisting movements. Moreover, spinal deformity imposes difficulties on multimodal image registration. Specifically, during image acquisition, patients can lay flatly for a short time due to pain, and subsequently, motion becomes unavoidable. Last but not the least, there are intrinsic differences between CT and MR imaging.

**Figure 1:**
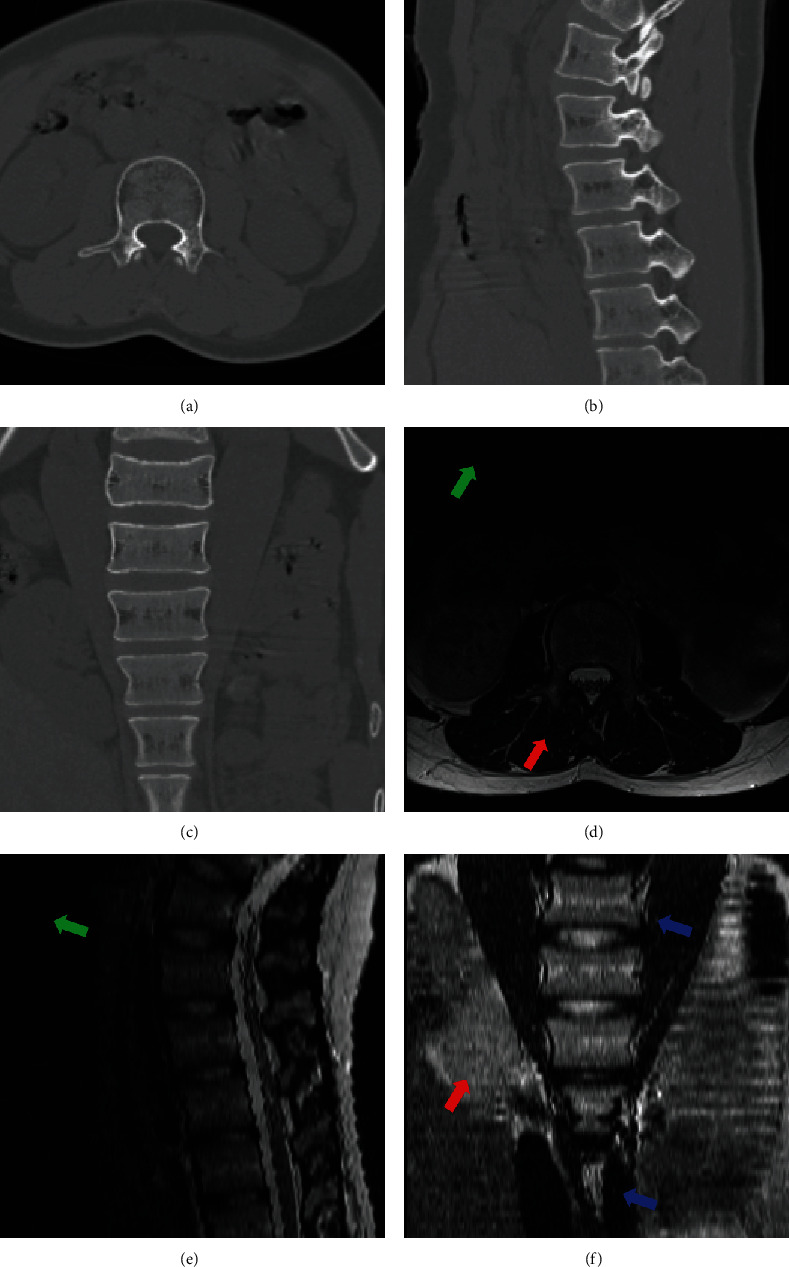
Perceived visual difference between CT and MR images of lumbar spine from three perspective views. The difference of imaging characteristics, fields of view, and unavoidable motion make the registration challenging. Red arrows show different imaging contrast, green arrows direct to the undesirable artifact of bias field in MR images, and blue arrows indicate different field of views. Note that images are cropped and scaled for display purpose.

Figure 1 shows a pair of intrapatient CT-MR images. It is found that in CT images, the lumbar spine region easily highlights itself from the rest of soft tissues (the top row), while in MR images, soft tissues show various intensities and in particular, it might be hard to distinguish rigid bones from soft tissues (the bottom row). In the figure, soft tissues in MR images are with various contrast than those in CT images (red arrows), undesirable artifacts caused by the bias field are observed in MR images (green arrows), and these pairs of images show different imaging field of views. It is obvious that these facets pose difficulties in image registration.

## 2. Related Works

Image registration is important in medical image analysis [12, 13, 14]. Based on similarity metrics, registration methods could be generally classified into intensity- and feature-based methods. Among the intensity-based methods, mutual information (MI) is well known, and it was primarily presented for MR breast image alignment [15]. Afterwards, the metric is used in multimodal medical image registration [16]. For specific applications, MI has been modified to enhance the performance of image registration. For instance, normalized MI (NMI) was proposed for invariant entropy measure [17], regional MI was implemented to capture volume changes when local tissue contrast varied in serial MR images [18], localized MI was designed for atlas matching and prostate segmentation [19], conditional MI was developed to incorporate joint histogram and intensity distribution for image description [20], self-similarity weighted *α*MI was presented for handheld ultrasound and MR image alignment [21], and MI was also advanced with spatially encoded information [22].

Feature-based methods aim to quantify detected landmarks with features for image registration. Ou et al. collected multiscale multiorientation Gabor features to weight mutual-saliency points for matching [23]. Zhang et al. used scale-invariant features and corner descriptors for lung image registration [24]. Heinrich et al. designed modality independent neighborhood descriptor (MIND) which extracted the distinctive structure in small image patches for multimodal deformation registration [25]. Via principal component analysis of deformation, a low-dimension statistical model was learned [26]. Toews et al. combined invariant features of volumetric geometry and appearance for image alignment [27]. Determined by the moments of image patches, a self-similarity inspired local descriptor was presented [28]. Jiang et al. designed a discriminative local derivative pattern which encoded images of different modalities into similar representation [29]. Woo et al. combined spatial and geometric context of detected landmarks [30], and Carvalho et al. considered intensity and geometrical features [31] into a similarity metric. Weistrand and Svensson constrained image registration with anatomical landmarks for local tissue deformation [32].

Embedding a proper penalty term into a similarity metric is helpful in specific applications. Rueckert et al. used a term to regularize the local deformation to be smooth in breast MR image registration [33]. Rohlfing et al. designed a local volume preservation constraint, assuming the soft tissues incompressible in small deformation [34]. Staring et al. proposed a rigidity penalty and modeled the local transform when thorax images with tumors were aligned [35]. To model fetal brain motion, Chen et al. utilized the total-variation regularization and a penalty was adopted toward piece-wise convergence [12]. Due to local tissue rigidity characteristics, Ruan et al. added a regularization term for aligning inhale-exhale CT lung images [36]. Fiorino et al. designed the Jacobian-volumehistogram of deforming organs to evaluate the parotid shrinkage [37].

This study proposes a coarse-to-fine framework to address the registration of intrapatient CT-MR images of lumbar spine. It develops a similarity metric that penalizes a bending energy term into NMI for local deformation of soft tissues. The most similar work is from the comparison of bending energy penalized global and local MI metrics in aligning positron emission tomography and MR images [38], while this study differs itself from the proposed coarse-to-fine registration framework, the bending energy penalized NMI (BEP-NMI) and the application to CT-MR lumbar spine images.

## 3. Materials and Methods

### 3.1. Data Collection

Two data sets were analyzed. One is our in-house collection which contains 40 pairs of lumbar spine images from the Department of Radiology, Shenzhen Second People's Hospital, the First Affiliated Hospital of Shenzhen University. CT images were acquired through SIEMENS SOMATO. The voxel resolution is 0.35 × 0.35 × 1.00 mm3, and the matrix size is 512 × 512 with 180 ± 25 slices. T_2_-weighted MR images were acquired using a 1.5 Tesla scanner (SIEMENS Avanto). The physical resolution is 0.7 × 0.7 × 3 mm3, the matrix size is 256 × 256, and the slice number ranges between 60 and 75.

The other data set is accessible online, namely SpineWeb (http://spineweb.digitalimaginggroup.ca). It includes 15 image pairs of lumbar spine. The physical resolution of CT images is 0.27 × 0.27 × 2.50 mm3, the image size is 512 × 512, and the slice number is 77 per volume. The resolution of T_1_-weighted MR images is 0.39 × 0.39 × 5.00 mm3, the image size is 512 × 512, and each volume contains 42 slices.

### 3.2. The Proposed Framework

The proposed framework consists of two steps both of which use intensity-based image registration methods. An intensity-based registration method can be treated as an optimization problem, and the similarity metric *S* performs as the cost function. Given a fixed image IF : *Ω*1 ∈ *R*3 and a moving image *IM* : *Ω*2 ∈ *R*3 in 3D space, image registration aims for mapping the moving image *IM* to the space of the fixed image IF guided by the metric *S*. When an additional regularization term of *P* is penalized into *S*, the registration problem can be formulated as,

(1) $$\begin{array}{c} \hat{\text{T}}=\arg\underset{T}{\min}\text{ C}\left(\text{T};{\text{I}}_{\text{F}};{\text{I}}_{\text{M}}\right)\text{ w}.\text{r}.\text{t}.\text{C}\left(\text{T}\mu ;{\text{I}}_{\text{F}};{\text{I}}_{\text{M}}\right)\\ \text{C}\left({\text{T}}_{µ};{\text{I}}_{\text{F}},{\text{I}}_{\text{M}}\right)=\text{S}\left({\text{T}}_{µ};{\text{I}}_{\text{F}},{\text{I}}_{\text{M}}\right)+\lambda \text{P}\left({\text{T}}_{µ}\right) \end{array}$$

where *T* is a transform model, *λ* compromises the metric *S* and the regularity term *P*, *μ* is the transform coefficients, and *Tμ* is the initialized model by *μ*.

**Figure 2:**
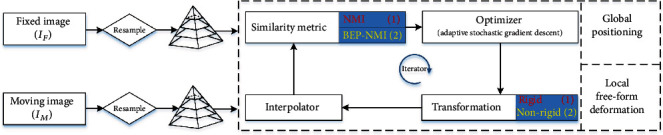
The proposed coarse-to-fine framework for aligning CT-MR lumbar spine images. It consists of two stages. The first stage is for global positioning via NMI based rigid registration (highlighted in red), and the second stage is for the local deformation of soft tissues via the bending energy penalized NMI (highlighted in yellow). Both stages utilize the same workflow for iterative optimization.

Figure 2 illustrates the proposed framework. It indicates a rigid registration stage and a hierarchical deformation stage, and NMI and BEP-NMI, respectively, perform as the similarity metric. Moreover, adaptive stochastic gradient descent (ASGD) [39] is applied for hyperparameter optimization. Specifically, an affine transformation with 12 degrees of freedom is employed in the first stage, and a B-spline elastic model is used for free-form deformation in the second stage.

#### 3.2.1. Rigid Registration

An affine transform model is used here. The transform *T* : *Ω*2 → *Ω*1 can be formulated by

(2) $$\begin{array}{c} {\text{T}}_{µ}\left(\text{x}\right)=\text{R}\left(\text{x}-\text{c}\right)+\text{t}+\text{c} \end{array}$$

where *R* is a matrix that contains the rotation, scale, and shear coefficients, *c* is the center of rotation, *t* is a translation vector, and *μ* is a vector of 12 degrees of freedom in volumetric image registration.

Rigid registration attempts for global positioning of the whole body, and thus, an initial alignment of lumbar spine. A 3-level recursive pyramid denotes smoothing that downsamples the source volumes by a factor of 2. Besides, the metric NMI and the affine transform are employed in each scale.

#### 3.2.2. Hierarchical Deformation

Hierarchical deformation is a coarse-to-fine adjustment procedure [40]. This setup utilizes Gaussian pyramid without downsampling to match images from the global structures toward the fine details.

B-spline transform. The B-splines are used to depict the local shape difference between the lumbar vertebrae. To construct the B-spline based free-form deformation model, let *Ω* = {(*x*, *y*, *z*) | 0 6 *x* < *X*, 0 6 *y* < *Y*, 0 6 *z* < *Z*} be a spatial domain of a 3D image. A lattice (*px* × *py* × *pz*) of control points is denoted as Ψ, spanning the integer grid in *Ω*, and *Φijk* denotes the control point at (*i*, *j*, *k*) on the mesh Ψ. Then, the elastic model can be expressed as a 3D tensor product of the uniform B-spline of order 3 as below,

(3) $$\begin{array}{c} T_{I}\left(x,y,z\right)=\overset{3}{\underset{I=0}{\sum }}\overset{3}{\underset{m-0}{\sum }}\overset{3}{\underset{n=0}{\sum }}B_{I}\left(u_{1}\right)B_{m}\left(u_{2}\right)B_{n}\left(u_{3}\right)\Phi_{i+l,j+m,k+n} \end{array}$$

where *i* = ⌊*x*/*P*_*x*_⌋ − 1, *j* = ⌊*y*/*P*_*Y*_⌋ − 1, *k* = ⌊*z*/*P*_*z*_⌋ − 1, *u*_1_ = *x*/*P*_*x*_ − ⌊*x*/*P*_*x*_⌋, *u*_2_ = (*y*/*P*_*y*_)⌊*y*/*P*_*y*_⌋, *u*_3_ = *z*/*P*_*Z*_ − ⌊*z*/*P*_*z*_⌋, and *Bl* repents the l^th^ basis function of the B-spline,

(4) $$\begin{array}{c} \left\{\begin{array}{c} B_{0}\left(u\right)={\left(1-u\right)}^{3}/6,\\ B_{1}\left(u\right)=\left(3u^{3}-6u^{2}+4\right)/6\\ B_{2}\left(u\right)=\left(-3u^{3}+3u^{2}+3u+1\right)/6\\ B_{3}\left(u\right)=u^{3}/6 \end{array}\right. \end{array}$$

where 0 6 *u* < 1. The basic functions weigh the contribution of each control point to *Tl*(*x*, *y*, *z*) based on its distance to the point (*x*, *y*, *z*).

Since the B-splines can be locally controlled, it makes the computation efficient for a large number of control points. In particular, changing a control point affects only the transforms of its local neighborhood.

BEP-NMI. The metric MI is preferred in multimodal image registration. Given IF and *IM* with intensity bins of *f* and *m*, MI is quantified from a joint probability function *p*(IF, *IM*) and marginal probability distribution functions.

of *p*(*f*) = *Pf* {*p*(*f*, *m*)} and *p*(*m*) = *Pm* {*p*(*f*, *m*)}. The metric MI between a pair of images, IF and *IM*, can be described as

(5) $$\begin{array}{c} \text{MI}\left({\text{I}}_{\text{F}};{\text{I}}_{\text{M}}\right)=\text{H}\left({\text{I}}_{\text{F}}\;\right)+\text{H}\left({\text{I}}_{\text{M}}\right)..\text{H}\left({\text{I}}_{\text{F}};{\text{I}}_{\text{M}}\right)=\underset{f\in F}{\sum }\underset{m\in M}{\sum }p\left(f,m\right)\log\langle \frac{p\left(f,m\right)}{p\left(f\right)p\left(m\right)}\rangle \end{array}$$

where *H*(IF) and *H*(*IM*) are the marginal entropy and the *H*(IF, *IM*) is the joint entropy of IF and *IM*.

The metric NMI is more robust to the change of overlapped tissue regions. It uses a Parzen-window approach to estimate the probability density function. The entropy of a fixed image IF is defined as *H*(*I*F) = −*Pf* ∈ *F* *p*(*f*)*logp*(*f*), where *p*(*f*) is a probability distribution estimated by using Parzen-windows. The entropy of a moving image *IM* can be computed in a similar way. And subsequently, the NMI between IF and *IM* can be presented as

(6) $$\begin{array}{c} \text{NMI}\left({\text{I}}_{\text{F}};{\text{I}}_{\text{M}}\right)=\frac{\text{H}\left({\text{I}}_{\text{F}}\;\right)+\text{H}\left({\text{I}}_{\text{M}}\right)}{\text{H}\left({\text{I}}_{\text{F}};{\text{I}}_{\text{M}}\right)} \end{array}$$

In order to regularize the B-spline deformation and to prevent the rigid structures from being smoothed, a BEP term *P*(*u*) is added to the NMI. The new cost function, BEP-NMI, is formulated as

(7) $$\begin{array}{c} \text{C}\left(\mu \right)={\text{y}}_{1}S\left(\mu \right)+y_{2}P\left(\mu \right) \end{array}$$

where *γ*1 and *γ*2 are predefined constants to weigh between global similarity and local regularity. In this study, off-line experiments indicated that *γ*1 = *γ*2 = 1 was a good choice.

The penalty terms are commonly based on the first or second-order spatial derivatives of the transform [35, 36]. In this study, the BEP term is composed of the second-order derivatives [35, 40] in the volumetric space,

(8) $$\begin{array}{c} {\text{P}}_{BEP}\left(\mu \right)=\int_{V}\left\{{\left(\frac{\partial^{2}T}{\partial x^{2}}\right)}^{2}+{\left(\frac{\partial^{2}T}{\partial y^{2}}\right)}^{2}+{\left(\frac{\partial^{2}T}{\partial z^{2}}\right)}^{2}+2{\left(\frac{\partial^{2}T}{\partial x\partial y}\right)}^{2}+2{\left(\frac{\partial^{2}T}{\partial y\partial z}\right)}^{2}+2{\left(\frac{\partial^{2}T}{\partial z\partial x}\right)}^{2}\right\}dxdydz \end{array}$$

where *V* is a 3D image. The Equation (8) can be approximated as a discretely sampled sum over the volume *V* as below,

(9) $$\begin{array}{c} {\text{P}}_{\text{BEP}}=\frac{1}{N_{V}}\underset{x\in V}{\sum }\Phi T\left(x,y,z\right) \end{array}$$

where *N* is the number of voxels in *V*, and *Φ* denotes a sum of the squared second-order derivatives of *T* inside the integral part in Equation (8) at a voxel location (*x*, *y*, *z*). Specially, the derivative approximation with finite differences can be restricted to the local neighborhood of the control point.

Optimization. Given an initial parameter *μ*, an optimization algorithm updates an incremental *∆μ* to reduce the cost function *C* iteratively. ASGD is used in the study, since it runs faster and less likely to get trapped in the local minima when compared to other gradient-based optimization algorithms [39]. Notably, ASGD implemented in the elastix package (http://elastix.isi.uu.nl) is used for adaptive step size prediction and the initial parameters are set as those in [39, 40].

### 3.3. A Comparison Method

The MIND is a feature-based method and it has been widely used in multimodal deformable registration [25, 41]. It aims to represent the distinctive image structure in a local neighborhood and explore the similarity of small image patches by using Gaussian-weighted patch distances [25].

MIND can be formulated by a distance *Dp*, a spatial search region *R* and a variance estimate *V* as below,

(10) $$\begin{array}{c} \text{MIND}\left(\text{I},\text{x},\text{r}\right)=\frac{1}{n}\exp\left(\frac{D_{p}\left(I,x,x+r\right)}{V\left(I,x\right)}\right)r\in R \end{array}$$

(11) $$\begin{array}{c} \text{Dp }\left(\text{I},\text{x}.\text{x}+\text{r}\right)=\text{C}*{\left(I-I\left(r\right)\right)}^{2} \end{array}$$

where *n* is a normalization constant, *r* the search region, *C* a convolution filter of size (2*p* + 1)*d*, ∗ a convolution filter, and *I*0(*r*) a dense sampling on *r*. As such, an image can be represented by a vector of size ∣*R*∣ at each location *x*. Moreover, *V* (*I*, *x*) can be computed based on a mean of the patch distances within a small neighborhood *n* (*n* ∈ *N*)

(12) $$\begin{array}{c} V\left(I,x\right)=\frac{1}{N}\underset{n\in N}{\sum }D_{p}\left(I,x,x+n\right), \end{array}$$

In Equation (10) to Equation (12), *n* = 6 denotes a six-connected neighborhood and *p* = 1 indicates a 3 × 3 × 3 volume block.

The similarity metric used in MIND comes from the sum of absolute difference. To the fixed image (IF) and the moving image (*IM*), the local difference at a voxel *x* is

(13) $$\begin{array}{c} LD\left(x\right)=\frac{1}{\left|R\right|}\underset{r\in R}{\sum }\left|MIND\left(I_{F,x,r}\right)-MIND\left(I_{M,x,r}\right)\right| \end{array}$$

The default value of ∣*R*∣ is 6 and it means 6-connected neighbors are taken into computation.

### 3.4. Performance Evaluation

#### 3.4.1. Tissue Overlapping

Tissue overlapping quantifies the overlapping ratio of outlined tissue regions in the fixed and its aligned image, which can distinguish the reasonable from the poor registration [42, 43]. This study focuses on the region of lumbar vertebrae and blood vessels. Assuming the outlined tissues in the fixed and aligned image are, respectively, denoted as OF and *OA*, the voxel-wise Jaccard (*J*) index and Dice (*D*) coefficient can be, respectively, described as

(14) $$\begin{array}{c} J=\left|\frac{O_{F}\cap O_{A}}{O_{F}\cup O_{A}}\right|,\\ D=2\frac{\left|O_{F}\cap O_{A}\right|}{\left|O_{F}\right|+\left|O_{A}\right|} \end{array}$$

where ∣·∣ indicates the number of voxels per volume.

#### 3.4.2. Target Registration Errors

As for landmark annotation, ImageJ (http://imagej.nih.gov/ij/) was used. A pair of CT-MR images are displayed side-by-side. Then, landmarks are identified and manually annotated by an imaging radiologist (3+ year experience) and further confirmed by a senior radiologist (10+ year experience). Once landmarks are annotated, their locations in 3D space are recorded. In this study, anatomical landmark points are localized on the vertebral body center (VBC), neural edge (NE), disc center (DC), and blood vessel edge (BVE).

Target registration error (TRE) evaluates the distance between anatomical point pairs in the fixed and moving image. Here, assuming *li* and , respectively, denotes the corresponding landmark point pairs in the fixed and moving image, the mean *TRE* for a given *T* is defined as

(15) $$\begin{array}{c} TRE=\frac{1}{n}\overset{n}{\underset{i}{\sum }}\left\|l_{i}-T\left(l_{i}^{\prime }\right)\right\| \end{array}$$

where *n* is the number of pairs of landmark, and *k* · *k* indicates Euclidian distance in 3D space.

### 3.5. Software and Platform

The whole framework is implemented with Insight Segmentation and Registration Toolkit (http://www.itk.org) and the elastix package [40]. Experiments are performed on a desktop computer equipped with dual-core Intel i7 CPU (3.70 GHz) and 16 GB RAM memory.

## 4. Results

### 4.1. Tissue Overlapping

**Figure 3:**
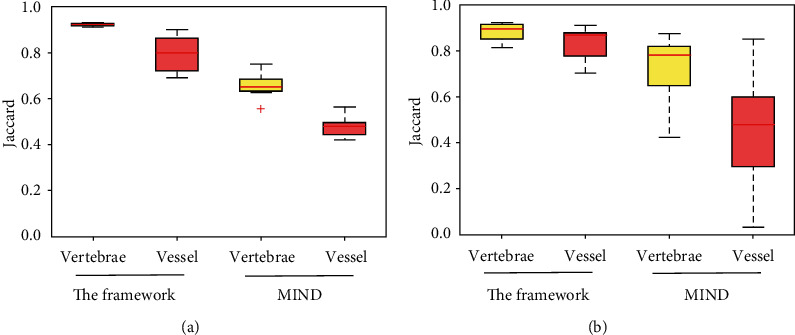
Jaccard index of the vertebrae and blood vessel overlapping on the in-house dataset (a) and the online dataset (b). Box-and-whisker plots represent the median Jaccard index (horizontal line) and total range (whiskers). The red ^+^ indicates an outlier that causes failure in image registration.

Figure 3 illustrates the tissue overlapping measure J of CT-MR image registration on the in-house collection (left) and the SpineWeb (right). The left shows that the proposed framework outperforms the MIND method on the vertebrae (0.93 ± 0.02 versus 0.69 ± 0.06) and blood vessel (0.81 ± 0.10 versus 0.48 ± 0.07) overlapping. In the right figure, the framework achieves higher values (vertebrae, 0.89 ± 0.05; blood vessel, 0.81 ± 0.12) than the MIND method (vertebrae, 0.75 ± 0.12; blood vessel, 0.52 ± 0.33), and thus, it leads to better performance.

**Figure 4:**
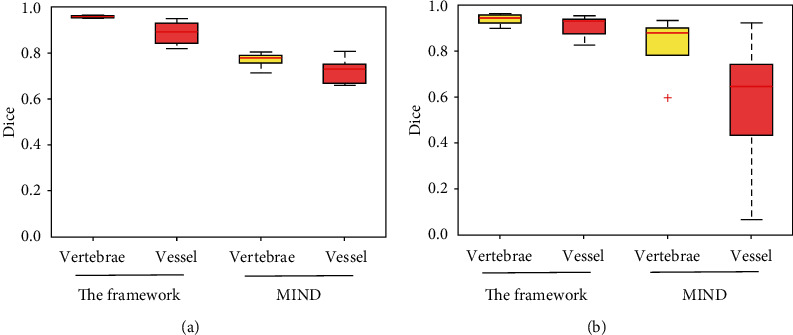
Tissue overlapping metric of Dice coefficient of the vertebrae and blood vessel on the in-house dataset (a) and the online dataset (b). Box-and-whisker plots show the median coefficient (horizontal line) and total range (whiskers). The red ^+^ indicates a failure case.

Figure 4 shows the overlapping ratio D of multimodal image registration on the in-house collection (left) and the SpineWeb (right). The left figure indicates that the coarse-to-fine registration framework obtains better results than the MIND method on the vertebrae (0.97 ± 0.02 versus 0.77±0.05) and blood vessel (0.88 ± 0.07 versus 0.74 ± 0.07) overlapping. In the right figure, the MIND method (vertebrae, 0.86 ± 0.12; blood vessel, 0.61 ± 0.33) obtains inferior performance than the proposed framework (vertebrae, 0.95 ± 0.03; blood vessel, 0.93 ± 0.10).

### 4.2. Target Registration Errors

**Figure 5:**
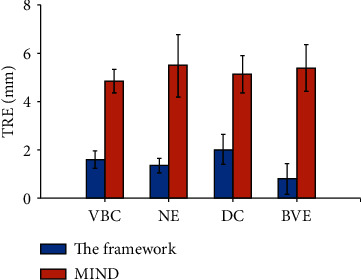
TRE values of anatomical landmarks on the in-house collection.

Figure 5 demonstrates the mean TRE value of anatomical landmark points between the proposed framework and the MIND algorithm on the in-house collection. The error-bar plot indicates that the TRE of the proposed framework is less than 3.00 mm (DC), while that of the MIND algorithm is larger than 4.00 mm (VBC) on average. In addition, statistical analysis indicates that the proposed framework significantly outperforms the MIND algorithm in each of the four sets of landmarks (*p* < 0.005, two-sample *t*-test).

Table 1 shows the TRE values (mean ± standard deviation, mean ± SD) with respect to different landmark sets. The coarse-to-fine framework achieves TRE between 0.78 ± 0.64 mm (BVE) and 2.01 ± 0.62 mm (DC), while the TRE of the MIND method ranges from 3.77 ± 4.21 mm (BVE) to 5.11 ± 3.69 mm (DC), correspondingly larger than that from the proposed framework.

**Figure 6:**
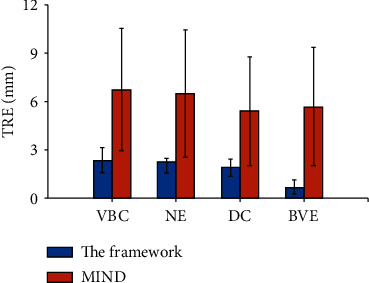
TRE values of anatomical landmarks on the SpineWeb dataset.

The mean TRE on the SpineWeb dataset is shown in Figure 6. It is observed that the TRE value of the proposed framework is less than 3.00 mm (VBC and NE), while the MIND algorithm leads to the TRE values larger than 5.00 mm.

Statistical analysis indicates significant difference of the TRE values between the proposed framework and the MIND algorithm on aligning the pairs of VBC and BVE landmarks (0.01 < *p* < 0.05, two-sample *t*-test).

Table 2 summarizes the mean TRE values on different sets of landmark pairs. The proposed framework achieves the TRE values between 0.66 ± 0.46 mm (BVE) to 2.37 ± 0.76 mm (VBC), and the TRE values of the MIND algorithm ranges from 5.71 ± 3.65 mm (BVE) to 6.75 ± 3.80 mm (VBC).

### 4.3. Perceived Quality of Image Alignment

**Figure 7:**
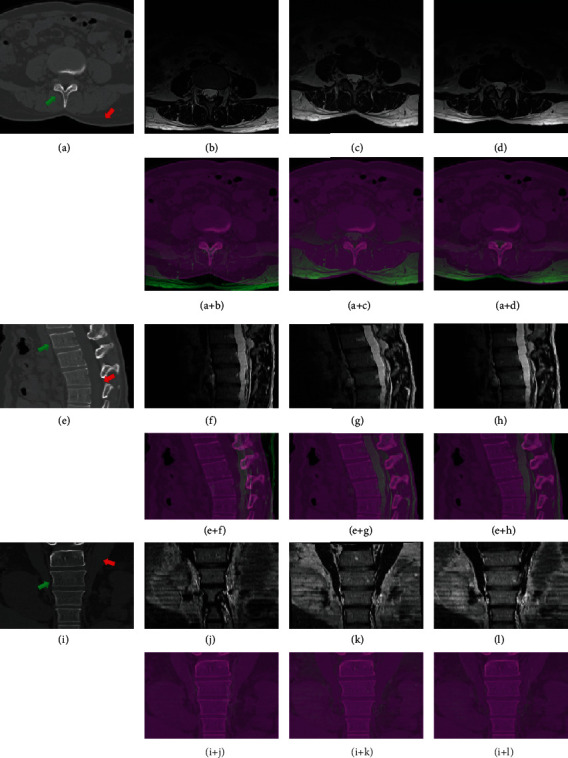
Perceived visual difference of CT-MR images before and after image registration. The regions directed by the arrows are for comparison before and after registration. In addition, images are cropped for display purpose.

Visual assessment of registration quality is perceived from the fusion of CT and MR images and observed from three perspective views in Figure 7, where (*A*, *E*, *I*) are the CT image, (*B*, *F*, *J*) are the MR image, (*C*, *G*, *K*) are the aligned image from the proposed framework, and (*D*, *H*, *L*) are the aligned image from the MIND algorithm. Red arrows directing to the soft tissue regions and green arrows directing to the bone regions are used for comparison. Before registration, both bones and tissues are misaligned, such as acantha (*A* + *B*), bones and nerves (*E* + *F*) and muscles (*I* + *J*). After image registration, the proposed framework aligns these parts in the MR images with fine deformation to the CT images. Specifically, both rigid bones and soft tissues are well matched, and the anatomical textures shows consistent distributions in the aligned image. On contrary, the MIND algorithm fails to overlap the acantha (*A* + *D*), bones and nerves (*E* + *H*) and muscles (*I* + *L*) accurately.

### 4.4. Computation Time

Based on the software and platform, it took about 62 seconds to complete the affine registration and 427 seconds to complete the deformable registration. And thus, it required a total of 8.15 minutes to fulfill the coarse-to-fine registration for a pair of CT-MR lumbar spine image.

## 5. Discussion

Intrapatient multimodal image registration can fuse multisource information that benefits disease diagnosis and treatment delivery. This study develops a coarse-to-fine framework and aligns intrapatient CT-MR lumbar spine images. It first utilizes the similarity metric NMI for global positioning, and then, bending energy penalized NMI for local deformation of soft tissues. The proposed framework achieves high tissue overlapping ratio and low target registration error. It not only preserves the incompressibility of vertebrae but also well matches local soft tissues that provide accurate elastic registration of lumbar spine images for clinical applications.

The proposed framework is a coarse-to-fine approach for multimodal image registration. It aligns anatomical structures and addresses the potential difference on the fields of view and the intrinsic differences between medical imaging. The metric NMI is used, since it is a robust and accurate measure in multimodal image registration [17, 44]. After global positioning, a new similarity metric that integrates a bending energy term into NMI is used for local deformation and registration of soft tissues in medical images. It is worth of note that the term encourages smooth displacements in registration [33]. Ceranka et al. embedded the term to improve multiatlas segmentation of the skeleton from whole-body MR images [45], and de Vos et al. integrated the term into unsupervised affine and deformable image registration by using a convolutional neural network [46]. Both works [45, 46] figured out that the term caused significantly less folding in image registration.

The framework takes the incompressibility of vertebrae into account. Vertebrae are bony structures which are connected to each other by the ligamentum flavum at the neural arch [47]. The proposed framework enables global and local image structures well matched, and inflexible bones and soft tissues properly deformed. Its superior performance has been verified on the in-house collection and the SpineWeb database. Experiential results demonstrate that the overlapping ratio of annotated vertebrae and blood vessels are larger than 0.85, and the target registration error is less than 2.40 mm on average. It outperforms the MIND algorithm partly due to its proper deformation of local soft tissues and incompressible lumbar vertebrae. The registration quality is further perceived in a CT-MR image pair. It is found that the marked tissues keep relative location after image registration by using the proposed framework, since it not only well tackles the local soft tissue deformation but also conserves the rigid lumbar vertebrae.

Even if the proposed framework achieves superior performance on aligning CT-MR lumbar spine images, there is still room for further improvement. One way to enhance registration accuracy is by transferring multimodal image registration into mono-modal image registration. Wachinger and Navab developed structural representations, such as Entropy and Laplacian images, which could represent the images in a third space where the images showed close intensity or gradient distribution [48]. Moreover, deep networks have been explored to estimate CT images from MR images directly and in particular, the mapping between CT and MR images was learned without any patch-level pre- or postprocessing [49]. Another straightforward way is to utilize deep networks to learn the deformation field between different imaging modalities [50]. In addition, interactive image registration is admirable in interventional surgery and a doctor user could localize landmarks to guide and to update the registration procedure [51].

There are several limitations in this study. One limitation comes from no comparison of NMI and BEP-NMI on deformable image deformation, since our off-line experimental results show that the NMI based deformable registration is prone to distortion of lumbar spine and unnatural deformation of soft tissues. Moreover, demons and its variants [52, 53, 54] failed in the registration of lumbar spine images. Thus, this study reports the performance of the proposed framework and the MIND method. In addition, how to properly balance the BEP term and the NMI is always a problem and no existing methods could well tackle this issue, while prior knowledge [35, 37] could be employed for further improvement of the registration accuracy.

## 6. Conclusions

This paper presents a coarse-to-fine framework for the registration of intrapatient CT-MR lumbar spine images. It integrates the bending energy term into normalized mutual information for fine deformation of soft tissues around the incompressible vertebrae. Its high performance benefits multisource information fusion for accurate spine disease diagnosis, treatment planning, interventional surgery, and radiotherapy delivery.

## Figures and Tables

**Table 1:** TRE values (mean ± SD) on the in-house collection images.

	The framework (mm)	MIND (mm)
VBC	1.52 ± 0.33	5.02 ± 3.76
NE	1.38 ± 0.29	5.07 ± 4.06
DC	2.01 ± 0.62	5.11 ± 3.69
BVE	0.78 ± 0.64	3.77 ± 4.21

**Table 2:** TRE values (mean ± SD) on the SpineWeb images.

	The framework (mm)	MIND (mm)
VBC	2.37 ± 0.76	6.75 ± 3.80
NE	1.91 ± 0.55	5.41 ± 3.38
DC	2.26 ± 0.98	6.49 ± 3.95
BVE	0.66 ± 0.46	5.71 ± 3.65

## Data Availability

The in-house collection of MR-CT image pairs used to support the findings of this study are restricted by the Medical Ethics Committee of Shenzhen Second People's Hospital in order to protect patient privacy. The SpineWebdata set of MR-CT images used to support the findings is freely available online (https://spineweb.digitalimaginggroup.ca/spineweb/index.php?n=Main.Datasets). If interested, requests for access to these data can be made to the author Shibin Wu (https://sb.wu@siat.ac.cn). Since the database is freely available, requests for access to these data can also be made to the author Shibin Wu (https://sb.wu@siat.ac.cn).
